# Neuromuscular electrical stimulation in critically ill traumatic brain injury patients attenuates muscle atrophy, neurophysiological disorders, and weakness: a randomized controlled trial

**DOI:** 10.1186/s40560-019-0417-x

**Published:** 2019-12-12

**Authors:** Paulo Eugênio Silva, Rita de Cássia Marqueti, Karina Livino-de-Carvalho, Amaro Eduardo Tavares de Araujo, Joana Castro, Vinicius Maldaner da Silva, Luciana Vieira, Vinicius Carolino Souza, Lucas Ogura Dantas, Gerson Cipriano Jr, Otávio Tolêdo Nóbrega, Nicolas Babault, Joao Luiz Quagliotti Durigan

**Affiliations:** 1 Health Sciences and Technologies PhD Program, University of Brasilia, Brasilia, DF Brazil; 2 Physical Therapy Division, Hospital de Base do Distrito Federal, Brasília, DF Brazil; 3 Rehabilitation Science Program, University of Brasilia, Brasília, DF Brazil; 4 Physical Therapy Division, Hospital Regional de Santa Maria, Brasilia, DF Brazil; 5 Escola Superior de Ciências da Saúde, Brasilia, Brasília, DF Brazil; 6 Clinical Research Center Hospital de Base do Distrito Federal, Brasilia, DF Brazil; 7 Science Health Program, University of Brasília, Brasília, DF Brazil; 8 Physical Therapy Department, Federal University of São Carlos, São Carlos, SP Brazil; 9 Centre de Recherche de l’Institut Universitaire de Gériatrie de Montréal, Montréal, Quebec Canada; 10 INSERM-U1093 Cognition Actionet Plasticité Senorimotrice, UFR STAPS, Université de Bourgogne-Franche-Comté, Dijon, France

**Keywords:** Critical care, Electrical stimulation therapy, Muscular atrophy, Muscle weakness, Neuromuscular diseases, Traumatic brain injury

## Abstract

**Background:**

Critically ill traumatic brain injury (TBI) patients experience extensive muscle damage during their stay in the intensive care unit. Neuromuscular electrical stimulation (NMES) has been considered a promising treatment to reduce the functional and clinical impacts of this. However, the time needed for NMES to produce effects over the muscles is still unclear. This study primarily aimed to assess the time needed and effects of an NMES protocol on muscle architecture, neuromuscular electrophysiological disorder (NED), and muscle strength, and secondarily, to evaluate the effects on plasma systemic inflammation, catabolic responses, and clinical outcomes.

**Methods:**

We performed a randomized clinical trial in critically ill TBI patients. The control group received only conventional physiotherapy, while the NMES group additionally underwent daily NMES for 14 days in the lower limb muscles. Participants were assessed at baseline and on days 3, 7, and 14 of their stay in the intensive care unit. The primary outcomes were assessed with muscle ultrasound, neuromuscular electrophysiology, and evoked peak force, and the secondary outcomes with plasma cytokines, matrix metalloproteinases, and clinical outcomes.

**Results:**

Sixty participants were randomized, and twenty completed the trial from each group. After 14 days, the control group presented a significant reduction in muscle thickness of tibialis anterior and rectus femoris, mean of − 0.33 mm (− 14%) and − 0.49 mm (− 21%), *p* < 0.0001, respectively, while muscle thickness was preserved in the NMES group. The control group presented a higher incidence of NED: 47% vs. 0% in the NMES group, *p* < 0.0001, risk ratio of 16, and the NMES group demonstrated an increase in the evoked peak force (2.34 kg/f, *p* < 0.0001), in contrast to the control group (− 1.55 kg/f, *p* < 0.0001). The time needed for the NMES protocol to prevent muscle architecture disorders and treat weakness was at least 7 days, and 14 days to treat NED. The secondary outcomes exhibited less precise results, with confidence intervals that spanned worthwhile or trivial effects.

**Conclusions:**

NMES applied daily for fourteen consecutive days reduced muscle atrophy, the incidence of NED, and muscle weakness in critically ill TBI patients. At least 7 days of NMES were required to elicit the first significant results.

**Trial registration:**

The trial was registered at ensaiosclinicos.gov.br under protocol RBR-8kdrbz on 17 January 2016.

## Background

Traumatic brain injury (TBI) is a frequent cause of morbimortality and represents a significant economic burden around the world [1, 2]. Mechanically ventilated critically ill TBI patients present a high risk of poor functional outcomes and often need substantial support after intensive care unit (ICU) discharge [3]. These patients demonstrate extensive muscle wasting, which occurs rapidly at the onset of a stay in the ICU [4]. In addition, patients can develop critical illness neuromyopathy, which is the leading cause of functional disorders [5]. This neuromyopathy alters nerve conduction and muscle excitability, inducing neuromuscular electrophysiological disorder (NED), which in addition to the muscle wasting, generates widespread muscle weakness [5]. The presence of NED is indicative of peripheral nerve disease with a sensitivity ranging from 90 to 100% [6]. The development of widespread muscle weakness among critically ill patients has been referred to as ICU-acquired weakness (ICUAW) [5, 7]. ICUAW patients also display high levels of plasma cytokines such as IL-6, IL-8, and TNF-*α*, which are associated with inflammatory and catabolic responses [8]. Clinically, ICUAW is associated with prolonged mechanical ventilation, longer ICU stays, and increased morbimortality rates [5]. Therefore, the prompt diagnosis of ICUAW is considered a cornerstone for preventing functional impairments [9].

Early rehabilitation in the ICU seems to be a feasible alternative for the prevention and treatment of ICUAW [10]. Among the treatments available for the early rehabilitation of patients in the ICU, neuromuscular electrical stimulation (NMES) has been considered a promising treatment [11]. Two systematic reviews concluded that NMES added to usual care proved to be more effective than usual care alone for preventing skeletal muscle weakness in critically ill patients [12, 13]. However, these studies found inconclusive evidence of its benefit in the prevention of muscle atrophy [12, 13]. In fact, there are particular gaps in the definition of a more efficient NMES protocol for non-cooperative critically ill patients [14, 15]. For example, the time needed for the NMES protocol to elicit the first countermeasure effects has still not been determined [16]. It appears that stimulation of a larger muscle area, as well as the production of maximum evoked contractions, is crucial for better results [16, 17]. Moreover, the number of stimuli per day and the number of treatment days could also be essential to generate significant results [18, 19]. Therefore, the present study aimed to assess the time needed and effects of an NMES protocol on muscle architecture, NED, and muscle strength, and, secondarily, to evaluate the effects on plasma systemic inflammation, catabolic responses, and clinical outcomes. The hypothesis was that the NMES protocol would counteract muscle atrophy and strength reduction, while preventing NED, and minimizing the presence of plasma inflammatory and catabolic responses.

## Methods

### Study design

This was a prospective, randomized, controlled, single-blind trial carried out over a period of 14 consecutive days. The study was performed in a neurotrauma ICU at a tertiary public reference hospital in the Federal District of Brazil. It was conducted according to the Declaration of Helsinki, and approval for the project was obtained from the local ethics committee (FEPECS/SES-DF, Brasília, Brazil, protocol 1.107.517). The trial was registered at the Brazilian Clinical Trials Registry (protocol number RBR-8kdrbz). The patient’s legal guardians signed an informed consent form since all patients were sedated or non-cooperative. The study is reported according to the Consolidated Standards of Reporting Trials and Statement for Randomized Trials of Nonpharmacologic Treatments and the Template for Intervention Description and Replication [20, 21].

### Randomization and allocation concealment

This was a 2-parallel group randomized clinical trial with a 1:1 intervention allocation. Computer-generated randomization lists were prepared using the website www.random.org, which sequentially distributed the patients into the control or NMES group. One researcher (PES) prepared sealed, opaque, and numbered envelopes. When each patient was enrolled in the study, the investigator opened the envelope with the smallest item number, containing the group.

### Blinding

A blinded researcher (KLC) completed all functional assessments (ultrasonography, NED, and evoked peak force) and gathered all clinical data on the electronic medical record of each participant. Plasma analyses were performed by another blinded researcher (VCS).

### Patients

Patients of both genders, between 18 and 60 years of age, who had undergone mechanical ventilation for up to 24 h, following a severe traumatic brain injury, were included. We excluded patients with a history of alcoholism, HIV, chronic kidney failure, spinal cord injury, pregnancy, skin lesions in the region to be treated, and patients with unstable fractures in the vertebral column and lower limbs.

### Study flow

Patients were randomized to the control or NMES group. From this time point, they were followed from the first 24 h of mechanical ventilation up to the 14th day. The assessment of muscle architecture, NED, evoked peak force, and plasma sample analyses were performed in both groups, after the first 24 h and on days 3, 7, and 14. Both groups were submitted to routine physiotherapy for early rehabilitation based on the protocol proposed by Morris et al. [22]. The physiotherapy routine protocol was applied for 10 to 30 min twice every weekday by the staff physiotherapists. In both groups, the level of routine physiotherapy and intensity were adapted to the patient’s cardiorespiratory status, level of sedation, cooperation, and functional status [22]. The protocol started with a global passive range of motion exercises in comatose or sedated patients, followed by active and resistive exercises, transfer to the edge of the bed or a chair, standing, and walking. The NMES group, in addition to daily routine physiotherapy, underwent NMES for 14 days bilaterally in the quadriceps femoris, hamstring, tibialis anterior, and gastrocnemius muscles.

### NMES protocol

NMES was applied using two identical electrical stimulator devices (Dualpex 071, Quark Medical, Piracicaba, Brazil). The electrodes were positioned according to the motor point, as previously described by Botter et al. [23]. Before initiating the NMES protocol, the criteria for starting and interruptions were followed, as proposed by Kho et al. [24]. The NMES was applied once a day for 25 min, with pulse duration and frequency of 400 μs and 100 Hz, respectively. The time on (T_ON_) was adjusted to 5 s and the time off (T_OFF_) to 25 s, thus eliciting a total of 50 contractions per day. The current amplitude was applied as high as possible to evoke maximum contractions in each muscle group (type 5/5, according to Segers et al. classification [25]).

### Outcomes

Primary outcomes were the effect of NMES over the muscle architecture, the presence of NED, and the evoked peak force. Secondary outcomes were the plasma level of cytokines and metalloproteinases, mechanical ventilation time, length of stay in the ICU, and length of hospitalization.

### Muscle architecture

Muscle architecture was assessed through muscle thickness and echogenicity using B-mode ultrasonography, with an ultrasound device, M-Turbo® (Sonosite, Bothwell, WA, USA). A water-soluble transmission gel was applied to the measurement site. A linear transducer of 7.5 MHz was positioned perpendicular to the tissue interface with the lowest possible skin compression. The muscle thickness was measured in two muscles: rectus femoris (RF) and tibialis anterior (TA). The transducer was positioned according to a previous recommendation by Arts et al. [26]. Evaluation of the RF was conducted at the mean distance between the anterior superior iliac spine and the superior border of the patella. The TA was evaluated at the proximal 1/4 of the distance between the inferior border of the patella and the lateral malleolus. Measurements were performed in the same predefined location during the intervention period. After acquisition of the images, the assessment of thickness was performed [26].

The RF thickness was measured between the upper part of the femur and the lower limit of the superficial fascia of this muscle since we only measured the RF thickness without the vastus intermedius muscle. We used the deep fascia of this muscle to delimitate the vastus intermedius muscle in order to exclude it.

The TA was measured between the interosseous membrane (on the side of the tibia) and the superficial fascia of the TA. Points were marked with a semi-permanent dermographic pen to avoid different positions over the days.

Muscle thickness and echogenicity were analyzed utilizing ImageJ software (http://imagej.nih.gov/ij/) [27]. Muscle echogenicity was measured through a quantitative grayscale analysis, where the most affected muscles had a white presentation (i.e., increased echogenicity). The echogenicity assessment area of analysis was selected in each muscle, including the maximum possible area (trace technique) [4] with an 8-bit image resolution, in values ranging from 0 (black) to 255 (white). The echogenicity and thickness were determined in each muscle, considering the mean value of the three different measures [26].

### Neuromuscular electrophysiological disorders

The presence of NED was assessed through the stimulus electrodiagnosis test (SET) in which rheobase and chronaxie were analyzed [4]. NED was recognized when chronaxie values reached ≥ 1000 μs [6]. Rheobase is the minimal current intensity necessary to reach the neuromuscular excitability threshold applied with a rectangular pulse with an infinite duration (e.g., 1 s). Chronaxie is defined as the shortest pulse duration required to reach the neuromuscular excitability threshold by a current with twice the intensity of the rheobase [4]. The rheobase and chronaxie were measured with a single-phase current and rectangular-shape current. For rheobase assessment, the intensity was increased from 1 to 69 mA with individual 1-mA increments until eliciting a slight and visible muscle contraction. The evaluation was performed with a pulse duration of 1 s and intervals of 2 s between pulses [4]. For the evaluation of chronaxie, the pulse duration was increased from 20 μs to 1 ms in increments of 100 μs. From 1 ms, increments of 1 ms were performed with a current amplitude twice the value of the rheobase until eliciting a slight but visible muscle contraction [4].

The SET was performed in two muscles: RF and TA. A reference electrode (anode), area 100 cm^2^, was placed on the patella for all measurements. The active electrode (cathode), in pen shape, approximately 1 cm^2^ in area, was used to find the motor points. The same electrode was used to determine the values of rheobase and chronaxie. The scanning area was established based on previous publications [23]. The location of the motor point was also marked with a semi-permanent dermographic pen.

### Evoked peak force

To evaluate the evoked peak force, we used a calibrated load cell (CKS model, Kratos Equipamentos, São Paulo, Brazil) attached to a platform and an electrical stimulator (Dualpex 071, Quark Medical, Brazil). Patients were laid down in a supine position with a 30° bed elevation. The platform was adjusted to the hip position at 90° of flexion and knee at 60° of the extension where the highest torque occurs [28]. The electrodes used to evoke muscle contraction were positioned on the RF muscle. The location was the line between the anterior superior iliac spine and the superior border of the patella at the motor points [23]. To find the motor point, we used a single-phase current of rectangular format with a pulse duration of 1 ms and 30 s of stimuli with an intensity of at least 10 mA. The anode electrode (100 cm^2^ of area) was placed on the patella and the cathode pen electrode (1 cm^2^ area) was used to perform the search for the motor point. Next, two electrocardiogram electrodes (≈ 1-cm^2^ area) were positioned on the motor points. The stimuli were performed on twitch contraction with 69 mA, T_ON_ of 3 s, pulse duration, and frequency of 400 μs and 100 Hz respectively. Three stimuli were performed, and the interval between each measurement was 2 min. We used the highest detected value among the measures.

### Clinical outcomes

In addition to the functional outcomes, clinical outcomes from medical records were analyzed as secondary outcomes. We evaluated time on mechanical ventilation, ICU mortality rate, length of stay in the ICU, and length of stay in the hospital.

### Plasma sample analysis

Approximately 12 mL of blood was collected from the antecubital vein by the standard venipuncture technique using a commercially produced vacuum-sealed kit. Tubes were centrifuged (Centrifugal machine, 3250RPM, Model Centurion, São Paulo, Brazil) at room temperature for 15 min at 2500 rotations per minute (≈ 1000×*g*). Serum was aliquoted (250 μL) and directly stored at − 80 °C until analyses by a blinded examiner. Serum levels of TGF-β and IGF-1 were obtained by regular enzyme-linked immunosorbent assays (ELISA). The circulating assessment of IL-1β, IL-6, IL-8, IL-10, and TNF-*α* was performed by a multiplexed flow cytometry method. The proteolytic activity was measured by analysis of metalloproteinases 2 and 9 activity using the zymographic method. Biological replicate samples of patients containing 1 μL of plasma were added to 1 μL of SDS (8%) (v:v). Metalloproteinases 2 and 9 activity were visualized as clear white bands against a blue background by densitometric scanning (ImageScanner III, Lab- Scan 6.0, Geneva, Switzerland). The analyses were performed in triplicate by a single-blinded examiner using ImageMaster 2D Platinum v7.0 (GeneBio) equipment, and the mean value of peak area was used in the final analysis (further details can be seen in Additional files 1 and 2).

### Statistical analysis

Data normality was tested with the Shapiro Wilk test, and parametric variables are described as mean and 95% confidence interval (95% CI). Nonparametric variables are presented as a median and interquartile range [IQR]. In order to measure the statistical differences in the continuous variables (chronaxie, evoked peak force, thickness, echogenicity, and biochemical variables), the two-way ANOVA (time × group) with repeated measurements was used followed by the Bonferroni post hoc test. To evaluate the categorical variables (presence or absence of NED determined by chronaxie ≥ 1000 μs) intergroups, Fisher’s exact test and log-Poisson regression to estimate risk ratio were used. The number needed to treat on day 14 of treatment was also computed. For the assessment of intragroup categorical variables, the McNemar test was used. Statistically significant differences were considered when *p* < 0.05. An intention-to-treat analysis was performed for all randomized participants. Missing data were replaced using the expectation-maximization method. For blood sample assessment, we evaluated an average of 10 participants per group due to an error in biochemical analysis. Thus, we present this outcome as a preliminary result. After each statistically significant comparison between groups, the effect size and power were calculated. Effect sizes were determined using partial eta squared (ηρ^2^). For the muscle architecture, NED, and evoked peak force data, where minimum clinically important differences were not nominated, Cohen’s *d* coefficient was calculated to aid interpretation. For this, Cohen provided benchmarks to define small (ηρ^2^ = 0.01), medium (ηρ^2^ = 0.06), and large (ηρ^2^ = 0.14) effects [29]. For statistical analysis, we used Statistica software, version 12 (StatsoftInc, Tulsa OK, USA, 2013).

Sample size was calculated using muscle thickness as the primary outcome. According to the study conducted by Gerovasili et al. [30], we estimated a difference between means and standard deviation of 1 mm ± 0.1 mm in muscle thickness after 14 days of treatment. Considering a study power of 85%, a significance level of 95%, and a sample size ratio of 1:1 (control group or NMES group), we reached the estimated number of 20 subjects per group on the 14th day. Thirty participants per group were recruited, totaling 60 subjects, allowing for possible dropouts during the intervention period [30, 31].

## Results

**Fig. 1:**
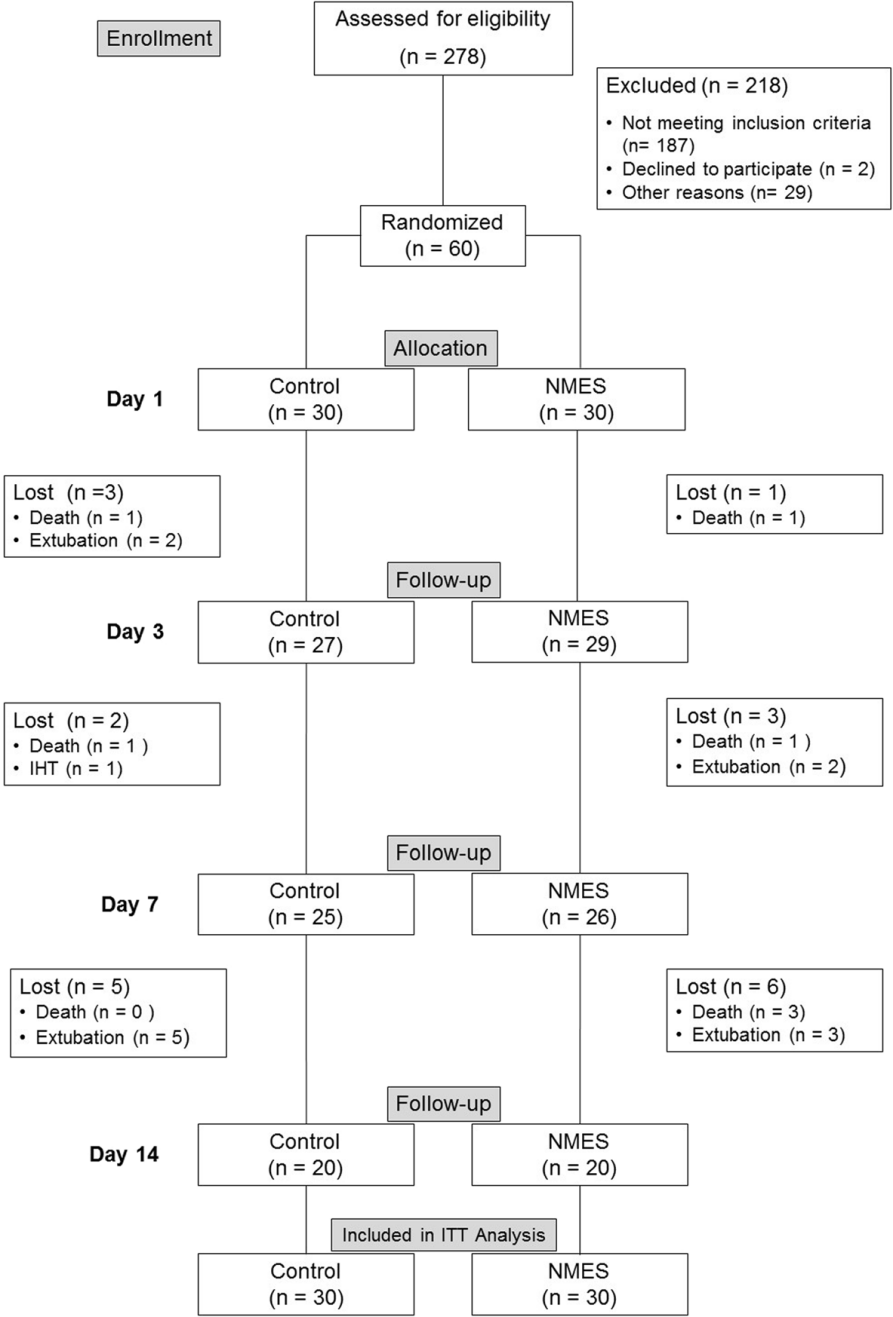
Consort diagram. IHT: inter-hospital transfers. ITT: intention-to-treat. Other reasons: technical problems, death before randomization, and inter-hospital transfers

Between June 2016 and July 2017, 278 patients with TBI were admitted to the Neurotrauma ICU, of these 60 were eligible according to the inclusion criteria and were therefore randomized for the study. The recruitment process and follow-up are described in the consort flow diagram (Fig. 1). Patient clinical characteristics are presented in Table 1. Intention-to-treat analysis was applied, and all patients were analyzed on the 14th day.

**Table 1:** Patient clinical characteristics

	Group	
Patient characteristics	Control	NMES
*n*	30	30
Age, years	33 (95% CI 29 to 37)	30 (95% CI 27 to 33)
Male sex, *n* (%)	26 (87%)	26 (87%)
AIS (head)	5 [5–5]	5 [5–5]
AIS (lower extremities)	1 [0–1]	1 [0–1]
Injury severity score	26 [26–30]	27 [26–34]
Cause of injury		
• Motorcycle, *n* (%)	11 (37%)	10 (33%)
• Motor Vehicle, *n* (%)	7 (23%)	2 (7%)
• Beating, *n* (%)	8 (27%)	3 (10%)
• Gunshot, *n* (%)	2 (7%)	6 (20%)
• Pedestrians, *n* (%)	1 (3%)	4 (13%)
• Fall, *n* (%)	1 (3%)	5 (17%)
Penetrating trauma mechanism, *n* (%)	3 (10%)	8 (27%)
Operative intervention, *n* (%)	20 (67%)	20 (67%)
APACHE II at ICU admission	11 [9–14]	11 [8–13]
SOFA at ICU admission	6 [4–9]	5 [5–8]
SAPS 3 at ICU admission	40 [32–47]	40 [30–48]
Diffuse axonal injury grade	2 [2–3]	3 [2–3]
Leucocytes on admission, unit	18.8 (95% CI 8.1 to 29.4)	16.7 (95% CI 14.5 to 18.9)
PaO_2_/FiO_2_ ratio on admission	296 (95% CI 260 to 331)	276 (95% CI 242 to 311)
Glucose over 14 days, mg/dl	144 (95% CI 130 to 158)	144 (95% CI 133 to 155)
Predicted enteral feeding, (%)	77 (95% CI 74 to 80)	79 (95% CI 75 to 83)
Use of vasopressor drugs, days	7 (95% CI 5.1 to 8.9)	7.7 (95% CI 6 to 9.4)
Use of corticoid drugs, days	0	0
Use of carbapenem antibiotics, n (%)	0	0
Days of sedation on ICU, days	10.8 (95% CI 9 to 12.5)	10.9 (95% CI 9 to 12.7)
Patients sedated on day 14, *n* (%)	19 (63%)	19 (63%)
RASS on day 14	− 3 [− 4 to − 3]	− 3 [− 5 to − 3]

*AIS* Abbreviated Injury Scale, *APACHE II* Acute Physiologic and Chronic Health Evaluation II, *ICU* intensive care unit, *SOFA* Sequential Organ Failure Assessment, *SAPS 3* Simplified Acute Physiology Score 3, *PaO*_*2*_*/FiO*_*2*_ ratio of arterial oxygen partial pressure to fractional inspired oxygen, *RASS* Richmond Agitation Sedation Scale. Parametric variables are reported as mean and (95% confidence interval) and nonparametric, as median and [interquartile range]

### NMES intervention

The quadriceps femoris, hamstring, tibialis anterior, and triceps sural muscles were stimulated at a mean intensity of 65 mA (95% CI 62 to 67). The general quality of evoked muscle contraction based on the Segers et al.’s [25] scale presented a median and [interquartile range] of 5 [4, 5]. From the initial fourteen expected NMES sessions per patient, eleven (95% CI 10 to 12) were performed on average, achieving a compliance rate of 79% (95% CI 68 to 84). Additionally, the mean intervention time of each session (electrode positioning and NMES protocol in all 4 muscle groups) was 72 min (95% CI 70 to 74). The main reasons for not performing NMES application were as follows: fever, 28 occurrences (46%), followed by hemodynamic instability, 19 occurrences (31%), psychomotor agitation, 9 occurrences (15%), and 5 sessions (8%) did not occur for other reasons.

### Complications

No cases of skin burn, or injury caused by NMES, occurred.

### Primary outcomes

#### Muscle architecture

**Fig. 2:**
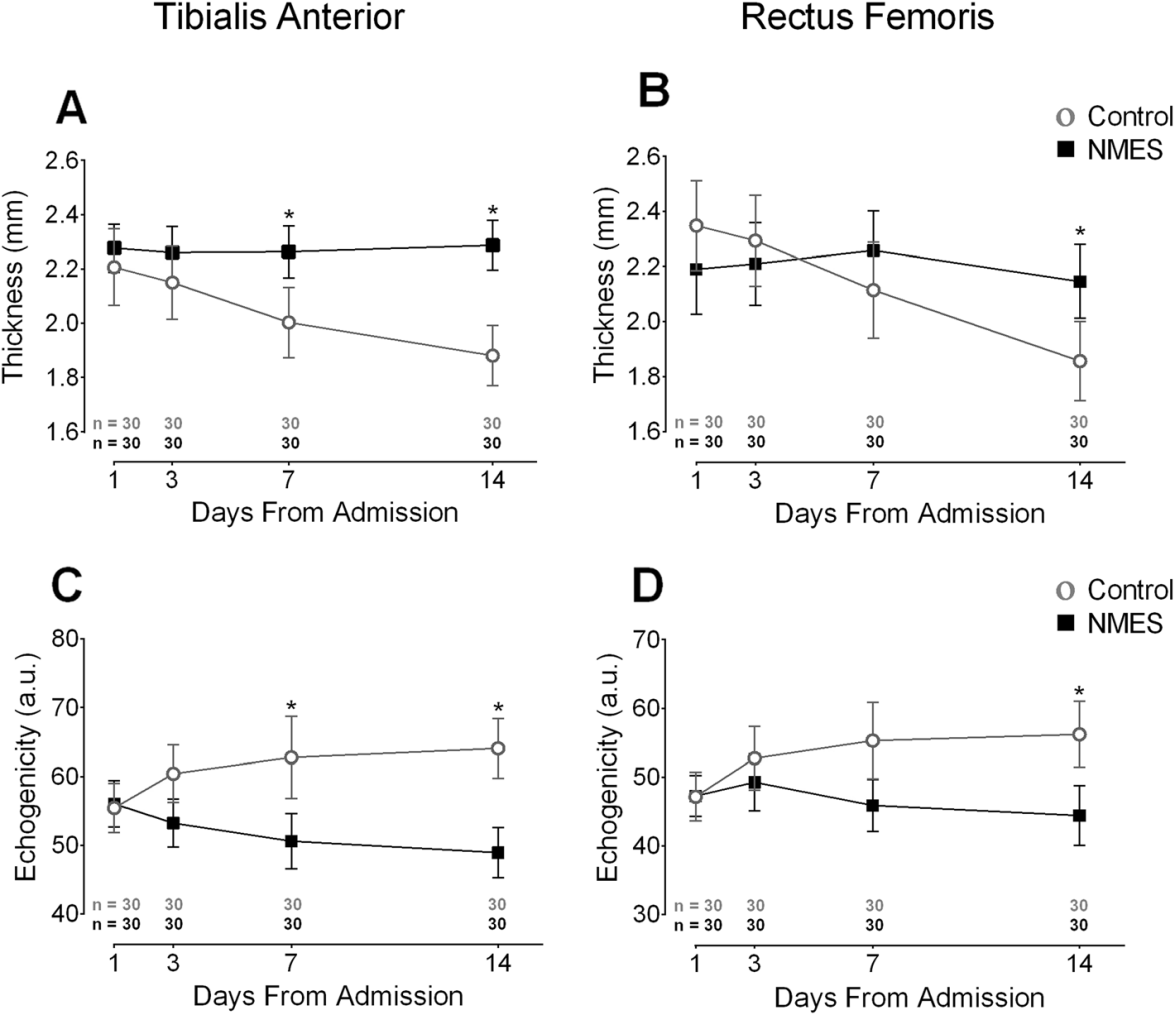
Effect of bed rest time and NMES on muscle architecture. The left graphs (**a** and **c**) present the tibialis anterior muscle architecture assessed by B-mode ultrasonography. On the right side (**b** and **d**), the rectus femoris muscle architecture assessed by the same test is presented. mm: millimeters; a.u.: arbitrary units. *: statistically significant time x group effect on highlighted day. This effect was analyzed by repeated measures two-way ANOVA. An intention-to-treat analysis was performed for all randomized participants

The comparison between groups over days demonstrated a statistically significant interaction in the TA in favor of NMES for preventing muscle loss: [interaction time × group (*F* = 30.9, *p* < 0.0001, power = 0.99, η_ρ_^2^ = 0.35)] (Fig. 2a). In the control group, the loss of muscle thickness _in _the _TA_ reached − 14% (95% CI − 17 to − 12) and − 0.33 mm (95% CI − 0.39 to − 0.26) on day 14, *p* < 0.0001. In the NMES group, muscle thickness did not significantly change on day 14 with a gain of 1% (95% CI − 4 to 3) and a mean difference of 0.01 mm (95% CI − 0.069 to 0.08), *p* = 0.78. The intraclass correlation coefficient (ICC) was calculated using three measures and showed excellent reliability (ICC 0.99) over the days. Similar results were found in the RF.

The comparison of muscle thickness between groups over days presented significant results in favor of NMES: interaction time × group [*F* = 29.9, *p* < 0.0001, power = 0.89, η_ρ_^2^ = 0.34] (Fig. 2b). The mean loss of RF thickness was − 21% (95% CI − 17 to − 24) and − 0.49 mm (95% CI − 0.58 to − 0.4) in the control group from baseline up to the 14th day, *p* < 0.0001. A non-significant loss was detected in the NMES group comparing the baseline with the 14th day, − 1% (CI 95% − 4 to 3) and − 0.04 mm (95% CI − 0.11 to 0.02), *p* = 0.15. The ICC was calculated using three measures and showed excellent reliability (ICC 0.98) over the days. NMES decreased the echogenicity of the TA and RF from the 7th and 14th days respectively, in the TA [interaction time × group (*F* = 17.1, *p* < 0.0001, power = 0.99, η_ρ_^2^ = 0.23)] (Fig. 2c), and the RF [interaction time × group (*F* = 18.4, *p* < 0.0001, power = 0.99, η_ρ_^2^ = 0.24)] (Fig. 2d).

### Neuromuscular electrophysiological disorders

**Fig. 3:**
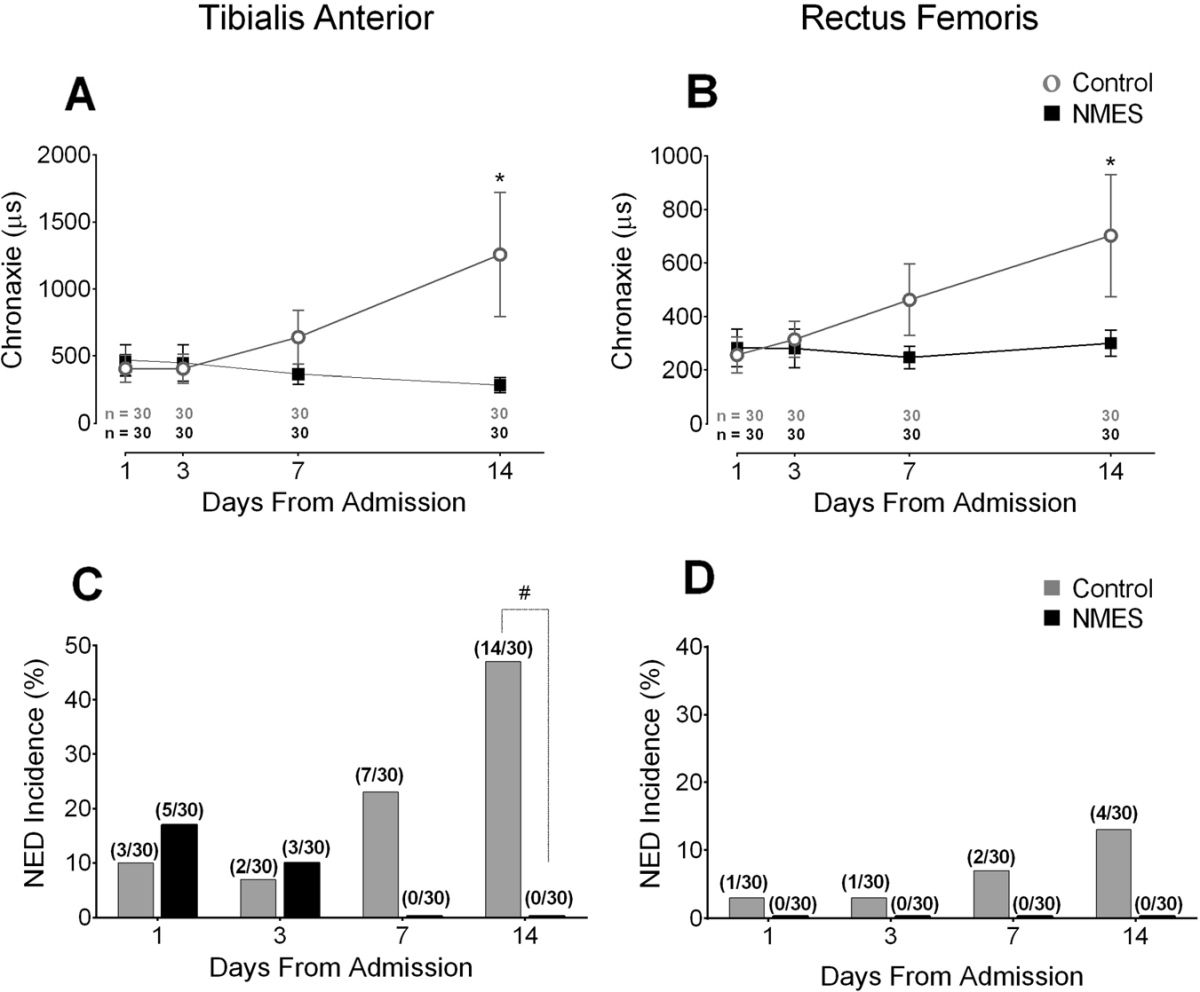
Effect of bed rest time and NMES on neuromuscular electrophysiology. The left graphs (**a** and **c**) show neuromuscular electrophysiology of the tibialis anterior assessed by the stimulus electrodiagnosis test. On the right side (**b** and **d**), the rectus femoris neuromuscular electrophysiology is presented, assessed with the same test. μs: microseconds; NED: neuromuscular electrophysiological disorder. *: statistically significant time x group effect on highlighted day. This effect was analyzed by repeated measures two-way ANOVA. #: statistically significant differences between groups in the NED incidence analyzed by the Fisher’s Exact test. The presence of NED was categorically defined once chronaxie ≥1000 μs. An intention-to-treat analysis was performed for all randomized participants

NMES induced significant reductions in chronaxie values in both the TA and RF. In the TA, significant differences were demonstrated between groups on day 14: [interaction time × group (*F* = 16.7, *p* < 0.0001, power = 0.99, η_ρ_^2^ = 0.22)] (Fig. 3a). In the control group, the TA chronaxie presented a significant increase over days: day 1 vs. day 14, *p* < 0.0001. NMES preserved neuromuscular excitability in the TA, maintaining chronaxie values over days: day 1 vs. day 14, *p* = 0.99. A similar significant interaction was observed for RF on day 14: [interaction time × group (*F* = 8.8, *p* < 0.0001, power = 0.99, η_ρ_^2^ = 0.13)] (Fig. 3b). In the control group, RF chronaxie values increased significantly over days: day 1 vs. day 14, *p* < 0.0001. In the NMES group, the neuromuscular excitability was preserved, demonstrated by chronaxie value maintenance over days: day 1 vs. day 14, *p* = 0.99. The control group presented NED incidence in the TA of 10% (3/30) on day 1 that increased to 47% (14/30) on day 14 (Fig. 3c), *p* = 0.003, power = 0.85. The NMES group presented NED incidence in the TA of 17% (5/30) on day 1 that decreased to 0% (0/30) on day 14 (Fig. 3c), *p* = 0.06. The control group presented a significantly higher incidence of NED (14/30) in the TA, compared with the NMES group (0/30) on the 14th day, (*p* = 0.0001, power = 0.99, and risk ratio = 16, (95% CI 2.9 to 88.9) (Fig. 3c). The control group also presented a higher incidence of NED in the RF than the NMES group on the 14th day: 13% (4/30) vs. 0% respectively, but this was not statistically significant *p* = 0.12 (Fig. 3d). Differences between groups were only detected at 14 days in the TA. Taking into consideration the NED incidence in the TA in both groups, the number needed to treat was 2.13 in 14 days of treatment to prevent a NED event.

### Evoked peak force

**Fig. 4:**
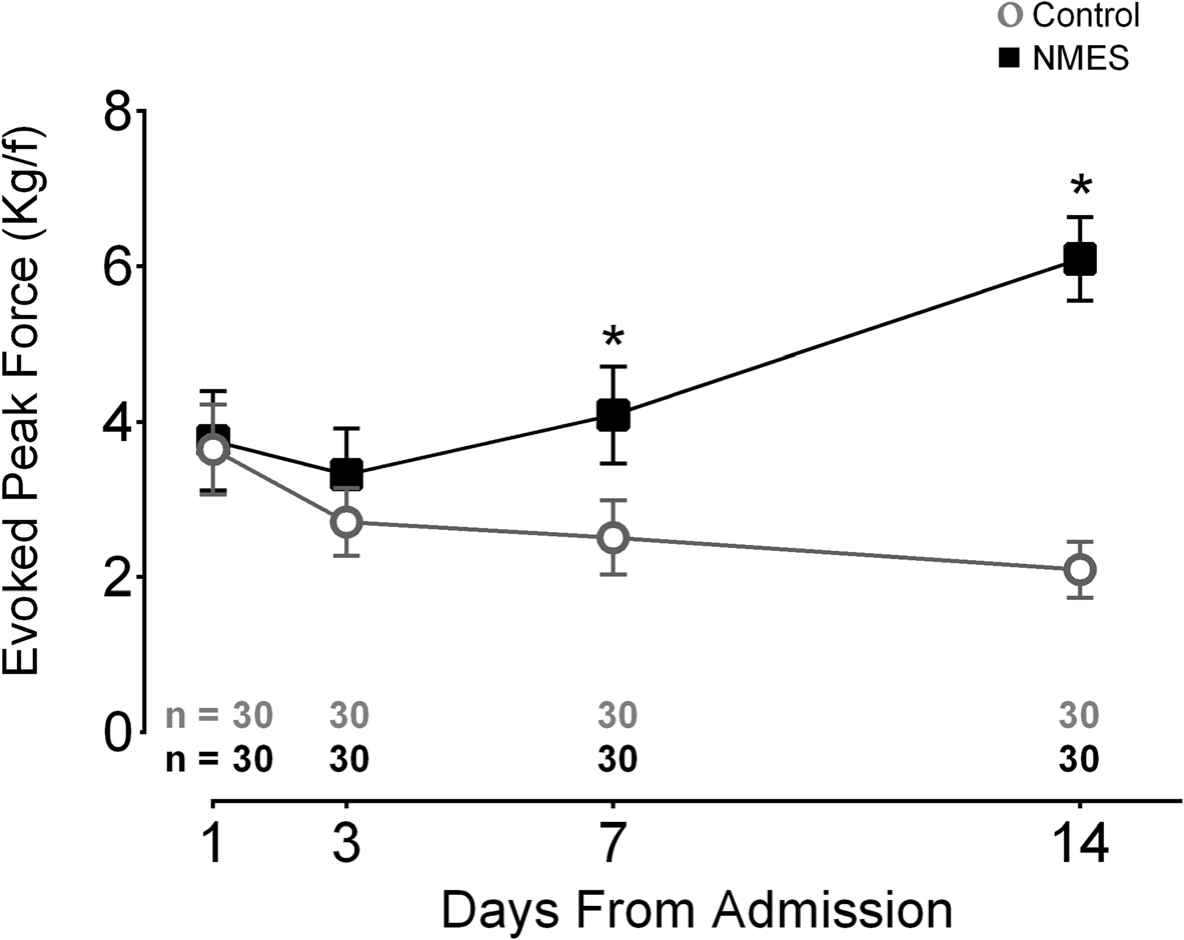
Effect of bed rest time and NMES on electrically evoked peak force. This graph presents the electrically evoked peak force of the rectus femoris muscle. The highest value after three bouts of electrical stimuli is reported. The contraction was elicited with a pulse duration and frequency of 400 μs and 100 Hz respectively with 69 mA amplitude and 3 seconds of time on. Two electrocardiogram electrodes were placed over the rectus femoris motor points. Kg/f: kilogram force; *: statistically significant time x group effect on highlighted day. This effect was analyzed by repeated measures two-way ANOVA. An intention-to-treat analysis was performed for all randomized participants

The comparison between groups over days demonstrated a statistically significant interaction in favor of NMES [interaction time × group (*F* = 71.9, *p* < 0.0001, power = 0.99, η_ρ_^2^ = 0.55)] (Fig. 4). Comparing with the baseline, patients in the NMES group presented a significant increase in evoked peak force from the 7th day, *p* = 0.001. In the NMES group, the evoked peak force increased from day 1 to day 14 with a mean difference of 2.34 kg/f (95% CI 1.89 to 2.79), *p* < 0.0001. On the other hand, the control group presented a significant decrement in evoked peak force from the 7th day compared with baseline, *p* < 0.0001. In the control group, the evoked peak force decreased from day 1 to day 14 with a mean difference of − 1.55 kg/f (95% CI − 2.05 to − 1.05), *p* < 0.0001. Differences between groups were detected from the 7th day, *p* < 0.0001.

### Secondary outcomes

#### Plasma sample analysis

The plasma cytokines (IGF-I; IL-1 β; IL-6; TGF-β; TNF-*α*) and metalloproteinases (MMP-2 and MMP-9) exhibited less precise results, with confidence intervals that spanned worthwhile or trivial effects. The data from these outcomes are presented in the Additional files 1 and 2.

#### Clinical outcomes

Patients in the control group remained on mechanical ventilation for 15.5 days [8.8–19] vs. 14 days [8–18] in the NMES group: median difference of 1.5 days, *p* = 0.65. The NMES group presented lower median differences in length of stay in the ICU (delta = − 0.5 day, *p* = 0.58) and hospital length of stay (delta = − 8 days, *p* = 0.06) but no significant statistical differences were detected. More details are presented in Table 2. No differences were detected in ICU mortality.

**Table 2:** Clinical outcomes

Group				
Outcomes	Control	NMES	*p* value	Effect size
*N*	30	30	–	
Incidence during the first 14 days, *n* (%)				
• Sepsis	13 (43%)	16 (53%)	0.44	–
• Septic shock	9 (30%)	10 (33%)	0.78	–
• Multiple organ failure	4 (13%)	6 (20%)	0.73	–
Time on MV, days	15.5 [8.8–19]	14 [8–18]	0.65	0.1
Time on MV (survivor), days	16 [9–19]	14 [12–18]	0.80	0.09
ICU length of stay, days	19.5 [12–27.3]	19 [10–26]	0.58	0.28
ICU length of stay (survivor), days	20 [15–31]	23 [15–26]	0.98	0.2
Hospital length of stay, days	42 [20–56]	34 [15–41.2]	0.06	0.5
Hospital length of stay (survivor), days	42 [23–53]	35 [23–44]	0.32	0.3
Mortality in ICU, *n* (%)	3 (10%)	5 (17%)	0.71	–

*ICU* intensive care unit, *MV* mechanical ventilation. Parametric variables are reported as mean and (95% confidence interval) and nonparametric, as median and [interquartile range]. *p* values were calculated by the unpaired *t* test, chi-square test, or Mann-Whitney in accordance with each data distribution and characteristics

## Discussion

The present study demonstrates that a clinical-like NMES protocol is effective to preserve the muscle architecture, increase evoked peak force, and decrease the incidence of NED. Muscle architecture and strength benefits were detected from the 7th day, while the effect of NMES to reduce NED was only observed from the 14th day of treatment. It seems that the time of NMES protocol needed is crucial to guide decision-making concerning treatment effects to counteract skeletal muscle atrophy, weakness, and NED in critically ill TBI patients. The present study was the first clinical trial to evaluate the effect of NMES on evoked peak force and neuromuscular excitability.

### Muscle architecture

Our results are supported by several studies that demonstrated the effectiveness of NMES to prevent muscle atrophy in critically ill patients [30, 32–34]. In a study with critically ill patients with similar clinical characteristics, Hirose et al. [33] showed that NMES prevented muscle atrophy in patients with consciousness disorders. These authors applied NMES for 42 days and demonstrated significant results in preventing muscle atrophy starting on the 14th day of treatment, in agreement with our results [33].

It seems that ICU admission etiology and clinical status are strongly related to muscle loss severity [35, 36]. Moreover, according to the study of Strasser et al. [34], the protective effect of NMES over muscle mass is correlated with the quality of evoked muscle contraction [34]. These authors compared the effect of maximum tolerable muscle contraction (~ quality type 5) with visible muscle contraction (~ quality type 3). Their results demonstrated a reduction in muscle atrophy only in the treatment with maximum tolerable muscle contraction.

Some studies [19, 31, 37] were not able to report an effect of NMES on muscle atrophy in the acute phase of critical illness. Gruther et al. [19], Fischer et al. [31], and Poulsen et al. [37] possibly used NMES protocols with lower intensities, since they reported evoking only visible contraction instead of reaching the maximum contraction, as has been recommended to induce muscle hypertrophy [17, 34, 38]. Additionally, Poulsen et al. [37] recruited extremely debilitated patients with septic shock who might not be able to benefit from this treatment [35].

### Neuromuscular electrophysiological disorders

We demonstrated that NMES can reduce the incidence of NED. The beneficial effects of NMES to treat NED may have been elicited through improvement in the neuromuscular and systemic circulation [39, 40]. The improvement in blood supply may protect neurons and myofibers against tissue dysoxia, which has been considered an important mechanism to induce axonal degeneration [39, 41]. Evoked contraction can also protect cellular machinery against disuse, mimicking physiological muscle contraction [32, 42].

Routsi et al. [43], in a landmark study, were the first to demonstrate the efficacy of NMES to prevent critical ill polyneuromyopathy, although without reporting therapeutic effects. A protocol for evoking 150 contractions was used with the current amplitude adjusted to elicit visible contraction (quality type from 3 to 4). In their study, the MRC scale was used to diagnose polyneuromyopathy.

In the present study, the presence of NED was used to define a diagnosis of peripheral nerve disease, which is expected in patients with polyneuromyopathy [44]. Paternostro-Sluga et al. [6] showed that the stimulus electrodiagnosis test (SET) is an excellent screening test to detect peripheral nerve disease with a sensitivity ranging from 90 to 100% when compared with needle electroneuromyography. Within the SET evaluation, we demonstrated a NED prevalence of 17% on the 1st day in the NMES group and an incidence of 10% on the 3rd day, though no cases were observed on the 7th and 14th days. Therefore, our results show that the current NMES protocol (fifty maximum evoked contractions) might not only prevent but also treat NED. Thus, the differences in NMES protocols and methods used to detect polyneuromyopathy may explain some discrepancies between the results of Routsi et al. [43] and ours.

### Evoked peak force

Muscle strength has been considered an independent factor for ICU mortality, length of stay, readmission to the ICU, and protracted function disability [12, 13, 45]. Therefore, we sought to assess strength through evoked peak force using an accurate and reliable new device as previously described [46]. Evoked peak force seems to be particularly advantageous over the MRC strength scale due to a higher sensitivity to detect change over time and the possibility of being used in unconscious patients [46, 47].

Even though we did not detect any increase in RF muscle thickness, the NMES protocol elicited a significant increase in evoked peak force compared with the control group. These findings are consistent with previous reports confirming that short periods of NMES can increase muscle strength even without hypertrophy [48]. It is now accepted that these strength gains are predominantly associated with neural adaptations [49, 50]. This idea is supported in the present study by lower levels of chronaxie identified in the NMES group. Chronaxie has been used to define the level of neuromuscular excitability, and typical values range from 60 to 200 μs [4]. If neuromuscular excitability decreases, chronaxie values increase [4]. It is important to emphasize that some events (such as sepsis and sedation) may impact muscle strength and should be considered when interpreting the present results [51].

In contrast, Fossat et al. [35] did not find any increments in muscle strength provided by NMES in critically ill patients. Considering the differences in the treatment protocol, their results could be also associated with the patients’ characteristics. In the present study, we controlled some treatment bias, as has been advocated by Reid et al. [52], comparing the effect of NMES solely with passive exercises. Moreover, Fossat et al. assessed muscle strength according to the MRC scale, which can present the ceiling effect bias [35].

### Plasma sample analysis and clinical outcomes

The estimates of the effect of the present protocol did not generate any clear implications about whether or not NMES plays a critical role in cytokines and metalloproteinases. Nevertheless, these preliminary data could support future randomized controlled trials. Despite the significant effect of NMES on functional outcomes (muscle architecture, NED, and evoked force), no statistically significant impact was found on the clinical outcomes: time on mechanical ventilation, length of ICU stay, and ICU mortality rate. These results may be associated with an insufficient sample size to detect a statistical difference for these secondary outcomes. Accordingly, a retrospective study with a large sample size (1118 neurocritical patients) demonstrated the significant impact of early rehabilitation for shortening ICU and hospital stays with a mean difference of 0.7 and 2.7 days respectively [53].

### Study limitations

Some limitations should be addressed in our study. This was a single-center trial with traumatic brain injury critically ill patients; thus, the findings may not be generalizable to different settings and patients. It was not possible to perform a follow-up of the primary outcomes, as stated in the CONSORT guideline. We did not assess muscle atrophy using the ultrasonography cross-sectional area. It is possible that our results underestimated muscle atrophy and missed statistical correlation with either of the outcomes, as recently described [54]. However, despite the higher sensitivity of the cross-sectional area compared with thickness, we were able to detect significant statistical differences with excellent reliability. In addition, although the appraiser was blinded to the groups, some healthcare providers were aware of the study allocation. Finally, the small simple size did not allow assessment of the effects of NMES on major clinical outcomes.

### Future perspectives

Further studies are required to define the optimal NMES prescription (parameters, number of contractions, therapy regularity, and treatment duration).

Furthermore, future multicenter trials should enroll an appropriate number of participants to better understand the effect of NMES on clinical outcomes. These studies should also evaluate the major clinical usefulness of NMES, such as the effect on treatment cost, ICU mortality, ICU length of stay, quality of life, and all domains of the International Classification of Functioning, Disability and Health (ICF) after hospital discharge.

## Conclusion

NMES applied daily for fourteen consecutive days reduced muscle atrophy, the incidence of neuromuscular electrophysiological disorders, and muscle weakness in critically ill TBI patients. At least 7 days of NMES were required to elicit the first significant results.

## Supplementary information

**Additional file 1.** Supplementary. Results.

**Additional file 2:** **Table S1.** Effect of NMES and bed rest on biochemical markers in critically ill patients over 14 days.

## Data Availability

The datasets generated during and/or analyzed during the current study are available from the corresponding author on request.

## Abbreviations

AIS: Abbreviated injure scale
ANOVA: Analysis of variance
CI 95%: 95% confidence interval
ICC: Intraclass correlation coefficient
ICU: Intensive care unit
ICUAW: ICU-acquired weakness
ISS: Injury severity score
NED: Neuromuscular electrophysiological disorder
NMES: Neuromuscular electrical stimulation
RF: Rectus femoris
SET: Stimulus electrodiagnosis test
TA: Tibialis anterior
TBI: Traumatic brain injury
ηρ^2^: Partial eta squared
