# Supplementary material for: Neuromuscular electrical stimulation in critically ill traumatic brain injury patients attenuates muscle atrophy, neurophysiological disorders, and weakness: a randomized controlled trial
Source: J Intensive Care. 2019 Dec 12;7:59. doi: 10.1186/s40560-019-0417-x (PMC6909464; doi:10.1186/s40560-019-0417-x)
Supplement: Supplementary file 1 — Additional file 1. Supplementary. Results. [file 40560_2019_417_MOESM1_ESM.docx]

**Method**

Approximately 12 mL of blood was collected from the antecubital vein by the standard venipuncture technique using a commercially produced vacuum sealed kit. Tubes were centrifuged (Centrifugal machine, 3250RPM, Model Centurion, São Paulo, Brazil) at room temperature for 15 min at 2500 rotations per minute (≈1000 × g). Serum was aliquoted (250 μL) and directly stored at −80 °C until analysed by a blinded examiner. *Inflammatory Biomarkers:* Serum levels of TGF-β, and IGF-1 were obtained by regular enzyme-linked immunosorbent assays (ELISA), using commercial kits from Quantikine® ELISA Human Immunoassay (R&D Systems, Inc., Minneapolis, USA) and an absorbance plate reader (ELx800, BioTek instruments, Inc., Winooski, USA). Procedures followed the manufacturer’s instructions, with thresholds of detection determined experimentally at 0.3 pg/mL for TGF-β and 15.0 pg/mL for IGF-1. Regarding circulating IL-1β, IL-6, IL-8, IL-10, and TNF-α, measurements were performed by a multiplexed flow cytometry method using the set of the Human Inflammatory bead-based immunoassay manufactured by BD Biosciences (San Diego, CA, USA), according to the manufacturer’s protocols. Briefly, serum samples were processed and the results acquired using the BD FACSCalibur flow cytometer, FL4 channel. Three hundred events were acquired for each cytokine bead used. Data were analyzed using FCAP software, version 3.0 (BD Biosciences, San Diego, CA, USA). Standard curves for each cytokine were generated using the lyophilized cytokine standards supplied, and the concentration was determined by interpolation from the corresponding standard curve. Detection thresholds were determined experimentally for each cytokine, standing at 5.0 pg/ml for IL-1β and IL-10, at 4.5 pg/ml for TNF-α, and at 4.0 pg/ml for IL-6 and IL-8. All standards and samples were measured in duplicate. *Zymographic analysis:* For the Zymographic analysis, biological replicate samples of patients containing 1μL of plasma was added to 1μL of SDS (8%) (v: v). Then the samples were vortexed and 10 μl of sample buffer without β-mercaptoethanol (reducing agent) containing SDS (20%) was added and resolved by polyacrylamide gel electrophoresis containing SDS and gelatin at the final concentration of 1mg/mL. After the run the gel was washed 2 times for 30 minutes in 2.5% solution of Triton X-100 to remove SDS. The gel was incubated in the substrate buffer (50 mM Tris-HCl pH 8.0, CaCl2.5 mM, 0.02% NaN3 and 10 mM ZnCl2) at 37°C for 20 hours. Next, the gels were stained with Coomassie brilliant blue R-250 for 1 ½ h and detained with acetic acid, methanol, and water. MMP-2 and MMP-9 activity were visualized as clear white bands against a blue background by densitometric scanning (ImageScanner III, Lab- Scan 6.0, Geneva, Switzerland). The analyses were performed in triplicate by a single blinded examiner using ImageMaster 2D Platinum v7.0 (GeneBio) equipment and the mean value of peak area was used in the final analysis.

**Results**

*Plasma sample analysis*

Time of bed rest and NMES did not promote any effect on IGF-I and TGF-β Interaction Time × Group (F = 0.7 p = 0.6, power = 0.18, η_ρ_^2^ =0.03) and Interaction Time × Group (F = 0.21 p = 0.9, power = 0.09, η_ρ_^2^ =0.01) respectively. The pro-inflammatory cytokines had the same outcomes: TNF-α [Interaction Time × Group (F = 2.3 p = 0.09, power = 0.55, ηρ2 =0.13)]; IL-6 [Interaction Time × Group (F = 0.17 p = 0.92, power = 0.08, ηρ2 =0.01)]; IL-8 [Interaction Time × Group (F = 0.9 p = 0.44, power = 0.23, ηρ2 =0.23)]; and IL-1β [Interaction Time × Group (F = 1.4 p = 0.25, power = 0.35, ηρ2 =0.07)]. Similarly, the anti-inflammatory cytokine IL-10 did not present any significant effect of time and NMES: IL-10 [Interaction Time × Group (F = 0.8 p = 0.5, power = 0.21, ηρ2 =0.05)]. More details are shown in Table S1.
