# Supplementary material for: Neuromuscular electrical stimulation in critically ill traumatic brain injury patients attenuates muscle atrophy, neurophysiological disorders, and weakness: a randomized controlled trial
Source: J Intensive Care. 2019 Dec 12;7:59. doi: 10.1186/s40560-019-0417-x (PMC6909464; doi:10.1186/s40560-019-0417-x)
Supplement: Supplementary file 2 — Additional file 2: Table S1. Effect of NMES and bed rest on biochemical markers in critically ill patients over 14 days. [file 40560_2019_417_MOESM2_ESM.docx]

**Table S1 - Supplementary Material**

Title: Effect of NMES and bed rest on biochemical markers in critically ill patients over 14 days.

| **Biochemical markers** | **Group** | **Time** | | | | | | | | |
| --- | --- | --- | --- | --- | --- | --- | --- | --- | --- | --- |
|  |  | **Day 1** |  | **Day 3** |  | **Day 7** |  | | **Day 14** |  |
| IGF-I (ng/ml) | Control | 136 (113 to 160) |  | 126 (92to159) |  | 161 (118 to 205) |  | | 174 (148 to201)^a^ |  |
|  | NMES | 106 (83 to 130) |  | 110 (74to 146) |  | 140 (90 to 191) |  | | 171 (123 to 219) ^a^ |  |
| TGF-β (ng/ml) | Control | 23 (15 to 30) |  | 25 (22 to 29 ) |  | 27 (18 to 35) |  | | 46 (37 to 55)^a^ |  |
|  | NMES | 24 (16 to 32) |  | 25 (18 to 32) |  | 32 (22 to 41) |  | | 47 (33 to 62) ^a^ |  |
| TNF-α (ng/mL) | Control | 9 (8 to 10) |  | 10 (9 to 11) |  | 9 (7 to 10 ) |  | 10 (9 to 11) | |  |
|  | NMES | 11 (7 to 14) |  | 9 (7 to 11) |  | 8 (7 to 8) |  | | 9 (8 to 11) |  |
| IL-6 (ng/mL) | Control | 233 (70 to 396) |  | 341 (75 to 606 ) |  | 239 (-110 to 597) |  | | 67 (26 to 108)^a^ |  |
|  | NMES | 188 (36 to 341) |  | 405 (-54 to 864) |  | 319 (-95 to 734) |  | | 134 (-30 to 298) |  |
| IL-1β (ng/mL) | Control | 18 (17 to 18) |  | 17 (17to18) |  | 18 (17 to 18) |  | | 18 (17 to 18) |  |
|  | NMES | 18 (17 to 19) |  | 18 (17 to 18) |  | 17 (17 to 18) |  | | 17 (17 to 18) |  |
| Active MMP-2 (a.u.) | Control | 0.21 (0.13 to 0.29) |  | 0.11(0.1 to 0.12) |  | 0.11 (0.09 to 0.13) |  | | 0.1 (0.08 to 0.12)^b^ |  |
|  | NMES | 0.10 (0.09 to 0.12) |  | 0.10 (0.09 to 0.12) |  | 0.08 (0.07 to 0.08) |  | | 0.08 (0.06 to 0.09)^b^ |  |
| Active MMP-9 (a.u.) | Control | 0.11 (0.1 to 0.12) |  | 0.11 (0.09 to 0.12) |  | 0.06 (0.05 to 0.08)^b^ |  | | 0.06 (0.05 to 0.07) ^b^ |  |
|  | NMES | 0.09 (0.1 to 0.13) |  | 0.08 (0.06 to 0.1) |  | 0.05 (0.02 to 0.09)^b^ |  | | 0.06 (0.05 to 0.07) ^b^ |  |

Legend: data are expressed as mean and 95% confidence interval. **IGF-I**: insulin growth factor; **IL-1 β**: interleukin-1 beta; **IL-6**: interleukin-6; **NMES**: neuromuscular electrical stimulation; **TGF-****β**: transforming growth factor beta; **TNF-α**: tumor necrosis factor alpha;**^a^:** statistically significant time effect;**^b^:** statistically significant interaction time × group effect. **MMP-2**: matrix metalloproteinase-2; **MMP-9**: matrix metalloproteinase-9; **a.u**.: arbitrary units; data were analyzed with repeated measure Two-Way ANOVA.
